# Synthesis and Effects of Two Novel Rare-Earth Energetic Complexes on Thermal Decomposition of Cyclotetramethylene Tetranitramine (HMX)

**DOI:** 10.3390/ma13122811

**Published:** 2020-06-22

**Authors:** Xuefang Cao, Zhixian Wei, Jiangfeng Song, Hedan Zhang, Yuanyuan Qu, Fei Xie

**Affiliations:** 1 School of Environment and Safety Engineering, North University of China, Taiyuan 030051, China; s1714035@st.nuc.edu.cn (X.C.); s1604037@st.nuc.edu.cn (H.Z.); 2 School of Science, North University of China, Taiyuan 030051, China; s1408060@st.nuc.edu.cn (Y.Q.); s1508053@st.nuc.edu.cn (F.X.)

**Keywords:** energetic complex, HMX, promoter, compatibility

## Abstract

In order to explore the effect of the energetic complex on the thermal decomposition HMX, two new rare-earth energetic complexes [La(tza)(NO_3_)_2_(H_2_O)_4_]*_n_* (**1**) and [Ce(tza)(NO_3_)_2_(H_2_O)_4_]*_n_* (**2**) (Htza = tetrazole-1-acetic acid) were prepared by a solvent evaporation method. The obtained products were structurally characterized by Fourier-transform infrared spectroscopy (FTIR), elemental analysis, powder X-ray diffraction (PXRD), single crystal X-ray diffraction (XRD), and thermogravimetric analysis coupled with differential scanning calorimetry (TG-DSC). In addition, the compatibility of complex **1** with cyclotetramethylene tetranitramine (HMX) was studied by DSC and FTIR, respectively. Structural analysis suggested that complex **1** exhibits an orthorhombic, P 2_1_ 2_1_ 2_1_ space group, and the La (III) ion was 10-fold coordinated in a distorted double-capped antiprism configuration. Complex **2** featured a one-dimensional, right-handed helical infinite chain. The effect of complexes **1** and **2** on the thermal decomposition of HMX was investigated by DSC, which revealed that complex **1** showed a slightly better effect than **2** on the thermal decomposition of HMX and released more heat. Furthermore, complex **1** had good compatibility with HMX, indicating that it may act as a combustion promoter for nitrate ester plasticized polyether (NEPE) solid propellant.

## 1. Introduction

Nitrate ester plasticized polyether (NEPE) propellant is a new type of crosslinked, high-energy, solid propellant which uses polyether polyurethane as a binder, nitrate esters as plasticizers, and is filled with a large amount of the high-energy oxidant, cyclotetramethylene tetranitramine (HMX) [1,2,3]. It also contains a combustion agent, stabilizer, and combustion promoter [4]. Here, HMX is used as the main component of the NEPE solid propellant, and a large amount of HMX increases the pressure index of the propellant, so improving the thermal degradation properties of HMX will significantly improve the combustion performance of NEPE [5,6,7]. Although the combustion promoter accounts for only a small proportion of solid propellants (about 2%–5%), it can greatly improve their combustion performance [8,9,10], so studying the type and performance of combustion promoters is highly important.

Energetic metal complexes are generally assembled with energetic ligands and metal ions, which are synthesized by different coordination techniques to give them certain spatial structures. They also have stable structures and superior thermal stabilities, suggesting they may be used in explosives [11]. On one hand, the energy released by the complexes can provide additional energy during the thermal decomposition of HMX or the combustion of NEPE. The energy can also be used to decompose more active metal oxides in situ to promote the combustion of NEPE, which would result in a lower pressure exponent and improvement in the combustion performance of the solid propellants [12,13]. Therefore, when used as combustion promoters that provided a platform for making various nanostructures, energetic metal complexes have very promising prospects [14,15,16]. This has led to the preparation of many energetic metal complexes, and their promoter properties for the thermal decomposition of HMX; hexahydro-1, 3, 5-trinitro-1, 3, 5-triazine (RDX); and ammonium perchlorate (AP) have been studied. Two kinds of Ni(II) salt complexes were synthesized using tetrazole derivative 5,5′-azotetrazole (AZT) as a ligand, which respectively lowered the temperature of the exothermic peaks of HMX and increased the heat of decomposition [17]. Two Cu(I)/Cu(II) coordination compounds [18] were shown to have an obvious effect on the thermolysis of HMX. It has shown that when HMX is mixed with LLM-105, fluoroelastomer, or powdered aluminum, respectively, the decomposition peak temperature decreased [19]. Two Cu(II) compounds [20] based on an azole ligand were shown to accelerate the thermal decomposition of RDX. A Ni(II) coordination compound based on pyridyl-triazole [21], and two Pb(II) compounds based on 3-(tetrazol-5-yl) triazol [22] were good candidates for use as promoters of AP thermolysis.

Among nitrogen-rich organic ligands of energetic complexes, tetrazolium heterocyclic complexes have many coordination points, which allow them to be coordinated as neutral molecules or as aprotic anions. This facilitates the formation of high-dimensional structures by connecting independent or low-dimensional coordination units. Moreover, tetrazole rings can act as hydrogen bond donors or acceptors, which help increase the density and stability of the complexes [23,24,25,26]. Four new rare earth polymers—[La(tza)_3_ (H_2_O)_2_·2H_2_O]*_n_*, [Pr(tza)_3_ (H_2_O)_2_·2H_2_O]*_n_*, [Nd(tza)_3_ (H_2_O)_2_·1.5H_2_O]*_n_*, and [Sm_2_(tza)_6_ (H_2_O)_5_·H_2_O]*_n_*—were synthesized by reaction of Htza with oxides of rare earth [27]. A series of lanthanide-based complexes—[Ln(atza)_2_(CH_3_OH)(H_2_O)Cl][Ln = Pr, Nd, Sm], [Ln(atza)_2_(H_2_O)_3_]Cl [Ln = Eu, Gd], and [Tb(atza)_2_(H_2_O)_4_]Cl—were synthesized by reaction of LnCl_3_·6H_2_O with 5-aminotetrazole-1-acetic acid (Hatza) under the alkaline condition [28]. Therefore, carboxylate tetrazole compounds are excellent ligands for the construction of novel versatile complexes. As an energetic ligand, Htza can coordinate with different metal atoms. Its N atom can strongly coordinate with transition metals, while its O atom coordinates with rare earth metal ions. Recently, our team has also reported a series of energetic tetrazole complexes, including [Co(tza)_2_]*_n_*, [Bi(tza)_3_]*_n_* and [Fe_3_O(tza)_6_(H_2_O)_3_]NO_3_ [29]. However, fewer compounds acted as additives to HMX are reported for rare earth metals.

The addition of La_2_O_3_ to the catalyst carrier Al_2_O_3_ has been shown to have a good effect on automobile exhaust [30]. Cerium oxides have been successfully used to catalyze the redox reaction of CO and N*_x_*O [31]. The main pollutants emitted by vehicles are CO and NO*_x_* complexes, which are very similar to the thermal decomposition products of HMX [32]. Thus, it is expected that La(III) and Ce(III) complexes may be good combustion promoters for NEPE. Therefore, in this study, Htza was used as a ligand, and La and Ce as central ions to construct two rare-earth energetic complexes.

The compatibility between the energetic additives and other propellant components is the key to making stable and practical propellants. Good compatibility is achieved when additives have good long-term stability and uniformity in a system. In contrast, unexpected explosions due to decomposition reactions may occur [33]. Therefore, the compatibility of new complexes and HMX should be investigated further. One of the most commonly used methods for analyzing the compatibility of energetic materials is differential scanning calorimetry (DSC). Since DSC is convenient, efficient, safe, and requires only small sample quantities, it has broad applications and is also a common method for judging the phase properties of energetic materials. In addition, some non-thermal techniques, such as Fourier-transform infrared spectroscopy (FTIR), have been frequently used to expand the understanding of thermal behavior that is not observed at room temperature [34,35]. One of the purposes of this study was to investigate previously unreported compatibility data for interactions between complexes and HMX using DSC combined with FTIR. Such information can assist in the design and optimization of future propellant additives.

## 2. Materials and Methods

The experimental materials were all analytical grade and commercially available. The purity of all reagents was verified by a Vario ELIII elemental analyzer (Elementar, Hanau, Germany). Samples for FTIR were analyzed on an imported spectrometer. Powder X-ray diffraction (PXRD) patterns were obtained using Cu Kα-ray using monochromatic graphite at room temperature. In this study, the thermal decomposition performance of complexes was evaluated in the range from 40–400 °C under flowing N_2_ at 20 mL·min^−1^.

### 2.1. Synthesis of [La(tza)(NO_3_)_2_(H_2_O)_4_]_n_ (**1**) and [Ce(tza)(NO_3_)_2_(H_2_O)_4_]_n_ (***2***)

A solution using acetonitrile solvent (5 mL) containing Htza (0.01281 g, 0.1 mmol) and La(NO_3_)_3_·6H_2_O (0.02165 g, 0.05 mmol), was filtered after the addition of four drops of distilled water. The filtrate was sealed with plastic wrap, punctured, and then slowly evaporated at room temperature. After two days, colorless prism crystals were collected. Yield: 50% (based on La). C_3_H_11_LaN_6_O_12_ (462.09); C 7.76 (calc. 7.79); H 2.51 (2.38); N 18.33 (18.18)%.

An acetonitrile liquor (10 mL) containing Ce(NO_3_)_3_·6H_2_O (0.0434 g, 0.1 mmol) was added dropwise to a mixture of acetonitrile (10 mL) containing Htza (0.0768 g, 0.6 mmol). The mixed solution was stirred for 1 h and then filtered. The filtrate was sealed with plastic wrap, punctured, and then slowly evaporated at room temperature, which was allowed to evaporate slowly at room temperature, and after two days, colorless prism crystals were collected. Yield: 80% (based on Ce). CeC_3_H_11_N_6_O_12_ (463.30); C, 7.96 (calc. 7.77); H, 2.12 (2.37); N, 18.38 (18.13)%.

### 2.2. X-ray Single-Crystal Diffraction (XRD)

Complexes were analyzed by a SMART-1000 X-ray diffractometer (BRUKER ASX, Karlsruhe, Germany). At 298 (2) K, the sample was scanned by Mo Kα ray (λ = 0.71073 Å) in the form of ω/2θ. All single-crystal structures were solved by direct methods and anisotropically refined using a full-matrix least-squares F^2^ method using SHELXTL-97. All non-H atoms were refined in a full-matrix anisotropic thermal parameters approximation. The H atoms of the ligands were obtained using a riding model, while disordered tza^−^ and NO_3_^−^ in complexes were refined by performing split and occupancies refinement.

### 2.3. Fourier-Transform Infrared Spectroscopy (FTIR)

Samples were analyzed by an FTIR-84005 spectrometer (Shimadzu, Tokyo, Japan) from the spectral region 4000–400 cm^−1^, with a resolution of 0.4 cm^−1^, using KBr pellets.

### 2.4. X-ray Powder Diffraction (PXRD)

Complexes were measured by graphite monochromatic Cu Kα ray with a tube pressure of 40 kV and a tube flow of 100 mA on a Rigaku D/max-rA X-ray diffractometer (Rigaku, Tokyo, Japan).

### 2.5. TG-DSC Thermal Analysis

Samples were analyzed on a Mettler Toledo GC10 TG-DSC (Mettler Toledo, DE, USA). Complex (0.7 mg) was added to a closed platinum crucible and measured under a flowing N_2_ atmosphere (20 mL·min^−1^) at a heating rate of 10 K·min^−1^.

### 2.6. Effect of the Energetic Complexes on Thermal Degradation of HMX

To evaluate how the energetic complexes facilitated the thermal degradation of HMX, HMX was mixed with the synthetic complexes at a 19:1 mass ratio. The amount of the promoter used in this experiment on the Mettler Toledo DSC823E was similar to the amount of a typical catalyst, which was about 5% (mass ratio).

### 2.7. Compatibility Test between HMX and Complex ***1***

Since complex **1** had a more pronounced effect on the thermal decomposition of HMX than **2**, the compatibility of complex **1** and HMX was then studied by DSC and FTIR [36]. A sample was obtained by mechanically mixing and grinding complex **1** with HMX at 1:1 mass ratio for 5 min to maximize all possible interactions between complex **1** and HMX.

## 3. Results and Discussion

### 3.1. Structure Descriptions

#### 3.1.1. Structures of [La(tza)(NO_3_)_2_(H_2_O)_4_]*_n_* (**1**)

**Figure 1:**
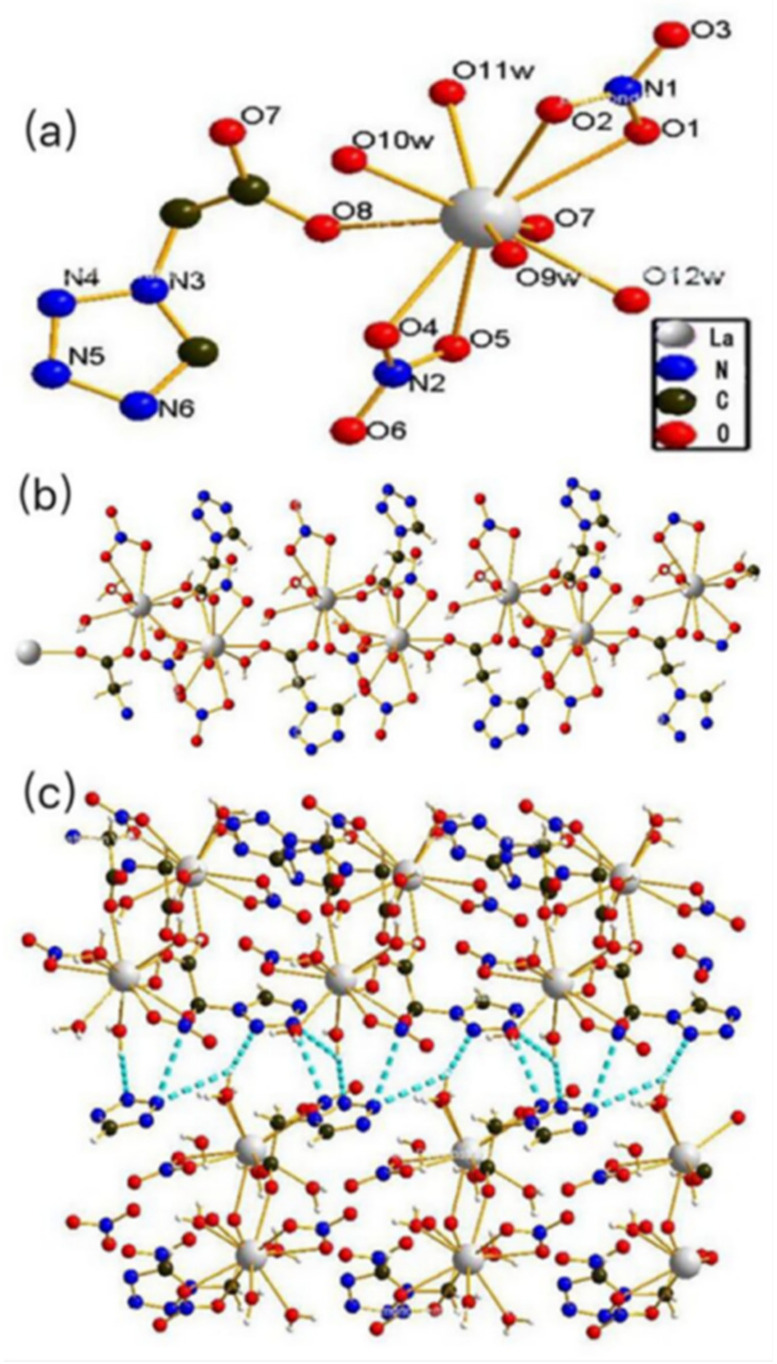
(**a**) The coordination environment of La(III); (**b**) one-dimensional chain of complex **1** with left-handed chirality; (**c**) three-dimensional network structure of complex **1** connected by O–H…N hydrogen bonds.

Complex **1** crystallizes in an orthorhombic space group P 2_1_ 2_1_ 2_1_. The asymmetric unit of the complex includes two independent La(III) ions, one tza^−^ ligand, two NO_3_^−^ ions, and four water molecules (Figure 1). The La(III) ion is 10-fold coordinated and located in a distorted double-capped antiprism configuration (Figure 1a), with the capping position occupied by two O atoms (O2 and O5) from two different NO_3_^−^ groups. One surface of the antiprism is composed of four O atoms (O7, O8, O9W, and O1) from two different tza^−^ groups, one coordinated water, and one NO_3_^−^ group. The other is composed of four O atoms (O10W, O11W, O12W, and O4) from three coordinated waters and one NO_3_^−^ group. The crystallographic data and detailed information of complexes are contained in Table S1, and selected bond information is shown in Supplementary Materials Table S2.

#### 3.1.2. Structures of [Ce(tza)(NO_3_)_2_(H_2_O)_4_]_n_ (**2**)

**Figure 2:**
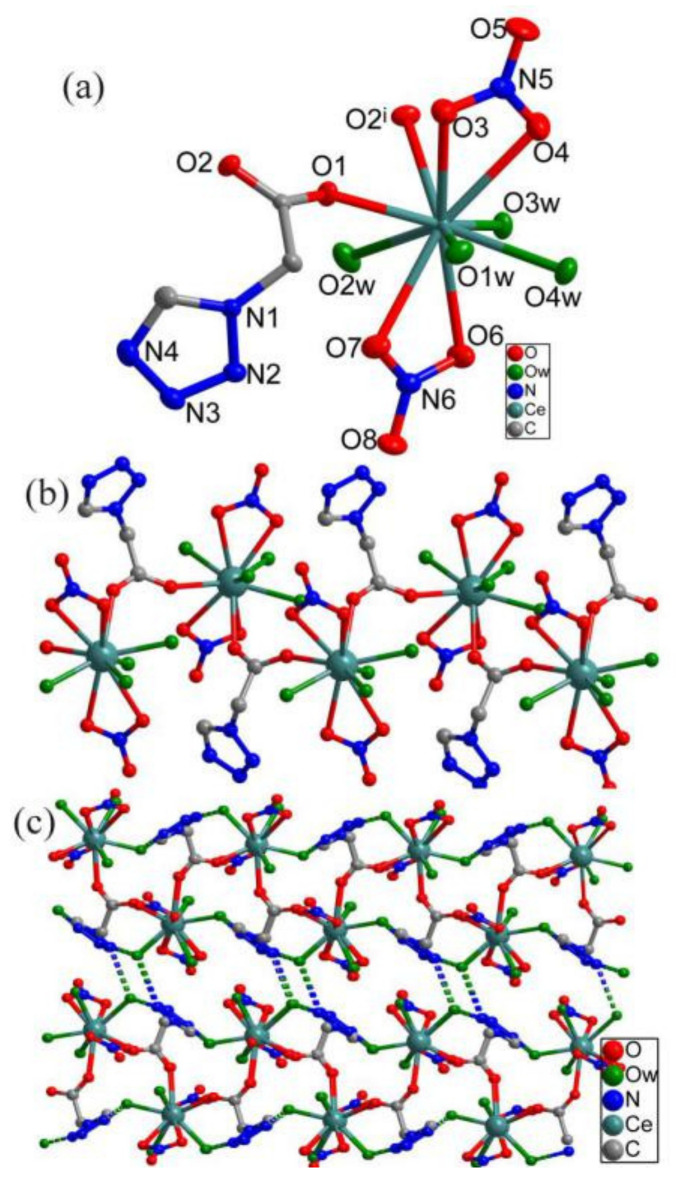
(**a**) The coordination environment of Ce with 50% ellipsoids; (**b**) one-dimensional chain with left-handed chirality; (**c**) three-dimensional network of **2** formed by O–H…N hydrogen bonds.

The asymmetric structure of complex **2** includes one Ce(III) ion, one tza^−^, two NO_3_^−^, and four coordinated water molecules. The Ce(III) ion is 10-fold coordinated and located in a distorted double-capped antiprism configuration (Figure 2a). The capping position is occupied by two O atoms (O3 and O6) from two different NO_3_^−^ groups. One square plane of the antiprism is formed by four O atoms (O1, O2, O1W, and O4) from two different tza^−^ groups, one coordinated water, and one NO_3_^−^ group. The other positions are occupied by four O atoms (O2W, O3W, O4W, and O7). The selected bond information is shown in Table S2.

As shown in Figure 2b, the tza^−^ anions connect with Ce^3+^ cations to form a 1-D infinite chain, which has a left-handed helical configuration along the b direction with adjacent Ce atoms separated by 6.10 Å. Each 1-D helical chain interacts with neighboring ones via O–H…N hydrogen bonds between water molecules and tza^−^ anions which leads to the formation of an extended 3-D supramolecular structure (Figure 2c). The corresponding bond information is listed in Table S2. Supplementary Materials of this article include X-ray crystallographic files in CIF reports, crystal data, and structure refinement for the complex, selected bond lengths, and bond angles in Supplementary Materials. CCDC no.18881025 for **1** and 1524937 for **2**.

### 3.2. Fourier-Transform Infrared Spectroscopy (FTIR)

**Figure 3:**
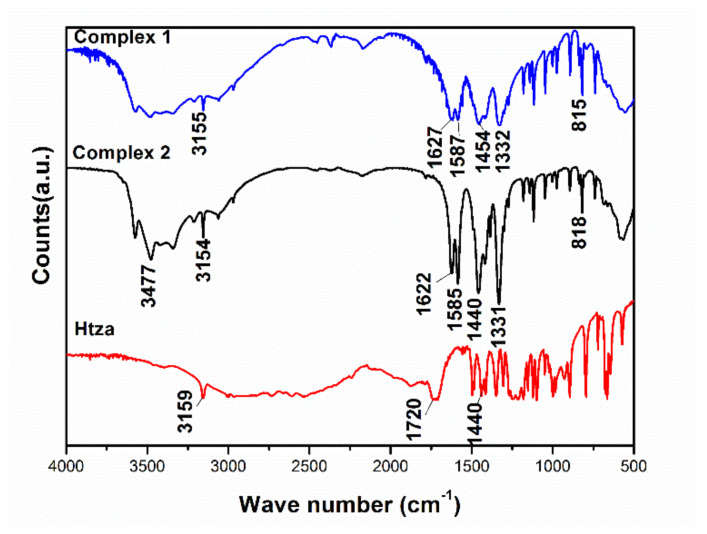
FTIR spectra of Htza, complex **1**, and complex **2**.

The FTIR spectrum of complex **1** has a wide characteristic absorption band from 3000 cm^−1^ to 3600 cm^−1^ (Figure 3), which corresponds to the O–H stretching vibration and the formation of hydrogen bonds between crystalline water and coordinated water in [La(tza)(NO_3_)_2_(H_2_O)_4_]*_n_*. The HOH bending vibration peak is at 1627 cm^−1^ [37]. The asymmetric stretching absorption vibration of Htza at 1720 cm^−1^ shifted to around 1600 cm^−1^, mainly due to the coordination of a La ion with an O atom in C(O)O^−^. Due to the symmetrical stretching vibration of C=O, an absorption peak can be seen near 1454 cm^−1^. The absorption peaks near 815 cm^−1^ appeared due to the in-plane bending vibration of –COOH (δ(C=O)). The absorption peak near 1332 cm^−1^ is the symmetrical vibration peak of –NO_3_ [38]. The above analysis is consistent with the synthesized crystal structure.

Since an O atom in C(O)O^−^ coordinated with a Ce ion, the stretching vibration of C=O in Htza shifted from 1720 cm^−1^ to around 1600 cm^−1^. The absorption peak near 1440 cm^−1^ appeared due to the symmetrical stretching vibration of C=O. The stretching vibration peak of –COOH was redshifted, which indicates that the C(O)O^−^ coordinated with Ce ions to form complex **2** [27]. The peak at 1622 cm^−1^ is due to the bending vibration of HOH [37]. The symmetrical absorption vibration peak of –NO_3_ is between 1300 and 1500 cm^−1^ [38] (Figure 3).

### 3.3. PXRD Diffraction Analysis

**Figure 4:**
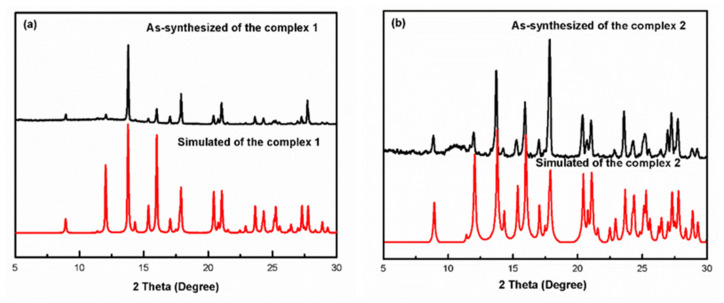
As-synthesized and simulated X-ray diffraction patterns of complexes **1** and **2**.

The purity of complexes **1** and **2** were analyzed by comparing the simulated diffraction data (the red one) with the experimental powder diffraction data (the black one). The simulated data and the experimental data of complexes **1** and **2** were consistent, respectively, indicating that the two complexes were both pure (Figure 4).

### 3.4. TG-DSC Thermal Analysis of Energetic Complexes ***1*** and ***2***

**Figure 5:**
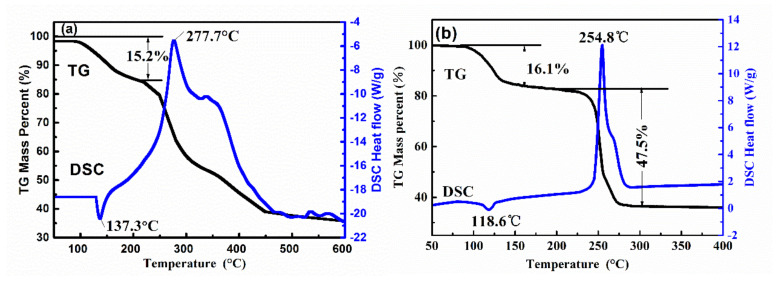
TG-DSC curves of complexes **1** (**a**) and **2** (**b**).

TG-DSC was used to investigate the thermal stability and degradation mechanism of complexes **1** and **2** (Figure 5). The TG curve of complex **1** is divided into three parts. During the first part, the weight loss ratio of coordinated water was 15.2% from 100 to 207 °C, which was similar to the theoretical value of 15.5%. In the second part, the tza^−^ ligands of complex **1** began to decompose, causing the complex framework to collapse between 207 °C to 450 °C. Significant mass loss occurred during this process, and it is inferred that the complex underwent a violent decomposition reaction within this temperature range. The frame of complex **1** collapsed, decomposed into solid products, and released gaseous products and heat. In the third stage, the TG curve was relatively stable from 450 to 600 °C. The whole process contained two distinct peaks in the DSC curve: one endothermic peak at 137.3 °C and one exothermic peak at 277.7 °C. Mass loss occurred due to the further decomposition of products from the second stage as the temperature continued to increase. The remaining 35.8% weight corresponded to the weight content of La_2_O_3_ (calcd. 35.3%). The decomposition peak temperature of complex **1** was 277.7 °C with a heat release of 1549 J·g^−1^.

The TG data of complex **2** shows that initial weight loss occurred from 79.1–164.1 °C. The weight loss during this temperature increase was about 16.1%, corresponding to a loss of four coordinated H_2_O molecules (calcd. 15.6%). Complex **2** continued to decompose with continuous heating because of the decomposition of NO_3_^−^. The weight loss process corresponded to the exothermic peak at 254.8 °C on the DSC curve. The remaining 36.4% of weight corresponds to the residual weight of Ce_2_O_3_, which is similar to the calculated value 35.4%. From Figure 5, it can be seen that the exothermic peak temperature of complex **2** is 254.8 °C, with a decomposition heat of 930.1 J·g^−1^. The experimental data reveals that both complex **1** and **2** have high decomposition temperatures and release high amounts of energy, indicating that they are thermally stable energetic complexes.

### 3.5. Effect of Energetic Complexes ***1*** and ***2*** on Thermal Degradation of HMX

**Figure 6:**
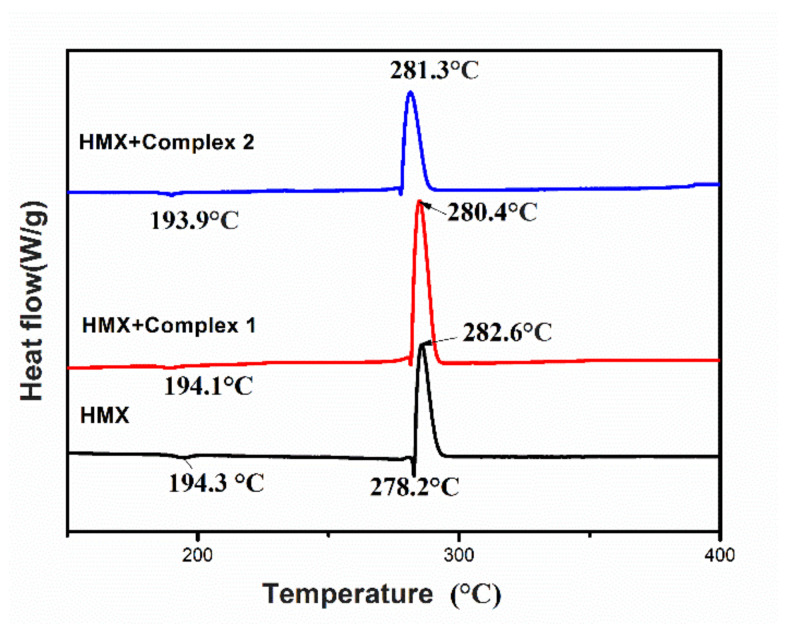
DSC curves of the HMX, HMX with complex **1** and HMX with complex **2**.

Complexes **1** and **2** were mixed with HMX at a 1:19 mass ratio to investigate how they affected the thermal degradation of HMX using DSC. From the DSC curve of pure HMX, it can be seen that the decomposition process contains two endothermic peaks and one exothermic peak (Figure 6). One of the endothermic peaks at 194.3 °C was attributed to the crystal transformation of HMX, while the other endothermic peak at 278.2 °C was due to the liquid phase degradation of HMX [39]. The exothermic peak occurred at 282.6 °C, with a heat release of 1198.3 J·g^−1^, which is nearly identical to the decomposition process of HMX reported in previous studies [18,40]. Energy can be obtained by integrating the area of Figure 6. By using the following formula, the actual increase or decrease of the heat release of the system can be obtained:

Δ*H* (J · g^−1^) = *H*_t_ − (5% × *H*_n_ + 95% × H_0_) (1)

where Δ*H* is the actual added heat release of the system, *H*_t_ is the total heat release of the mixed system, *H*_n_ is the heat release of the complex 1 or 2 and *H*_0_ is the heat release per gram of HMX.

However, the effects of compound **1** and **2** for HMX thermal decomposition could be explained as follows: (1) In the thermal decomposition processes of HMX with **1** and **2**, respectively, fresh metal oxides at the molecule level can be formed (Figure 5). The in-situ formed oxides with a high specific surface have high density of active sites on the surface and can play an important catalytic role. On the other hand, those fresh metal oxide powders would trap HMX as more adsorption sites formed. Such effects would make HMX less stable and result in the decrease of the decomposition peak temperatures of HMX and HMX mixtures. Furthermore, the formed fresh metal oxides can also adsorb more pyrolysis products of HMX and lead to the release of the adsorption heat [41]. (2) The main gaseous products of HMX are CH_2_O, NO_2_, CO_2_, NO, CO, and N_2_O [42,43,44]. It is noted that the thermal decomposition products of HMX are similar to those of automobile exhaust. Owing to La_2_O_3_ and Ce_2_O_3_ being able to catalyze the reactions of carbon oxides and nitrogen oxides in automobile exhaust [30,31]. One can conclude that a serious of exothermic reactions between the different gaseous products could be catalyzed with those fresh metal oxide mixtures, including the oxidation reaction of CO, the reaction between CO and NO, and so on. That could lead to the increase of heat release.

Compared with the DSC curve in Figure 6, the position of the first endothermic peak of HMX was nearly unchanged after complex **1** was added, which indicates that the addition of complex **1** had no effect on the crystal transition process of HMX. As an additive, complex **1** not only reduced the exothermic peak temperature of HMX by 2.2 °C, but also increased the heat release of HMX by 129.9 J·g^−1^ (Table 1). Therefore, the complex could promote the thermal degradation of HMX. When complex **2** was added, the exothermic peak temperature of HMX increased 1.3 °C, and the amount of liberated heat increased by 41.5 J·g^−1^. In addition, as an HMX addictive, the decomposition peak temperature reduction and heat release of the La complex (complex **1**) is more and higher than that of some transition metal complexes, such as [Ag(tza)]*_n_* (**3**), [Cu(tza)_2_]*_n_* (**4**) and [Zn(tza)_2_]*_n_* (**5**) [45], under the same conditions.

Since the two energetic complexes lowered the exothermic peak temperature of HMX and increased the amount of heat released, each can act as accelerators for the thermal decomposition of HMX. This suggests they may also be excellent combustion promoters of NEPE. Complex **1** exhibits a slightly better effect on the thermal degradation of HMX than **2** and lowers the decomposition temperature of HMX more and releases more heat under the same experimental conditions. Therefore, in this study, the compatibility between HMX and complex **1** was chosen for further study.

### 3.6. Compatibility between HMX and Complex ***1***

#### 3.6.1. DSC Analysis

**Figure 7:**
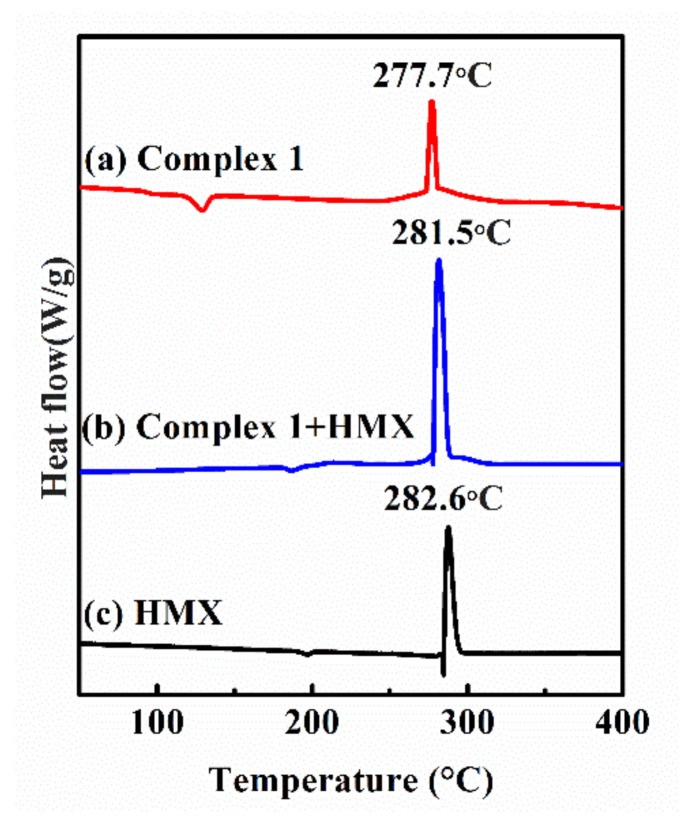
DSC curves for complex **1** (**a**), complex **1** and HMX (**b**), and pure HMX (**c**).

As shown in Figure 7, the difference between the exothermic peak temperature (Δ*T*_p_) of HMX and the mixture of complex **1** and HMX is 1.1 *°*C (i.e., less than 2 °C). According to the compatibility evaluation criteria of explosives and contact materials in Table 2, it is known that complex **1** has good compatibility with HMX and is safe for use in any explosive design when the exothermic peak temperature difference (Δ*T*_p_) is less than 2 °C [46,47,48].

#### 3.6.2. FTIR Method

**Figure 8:**
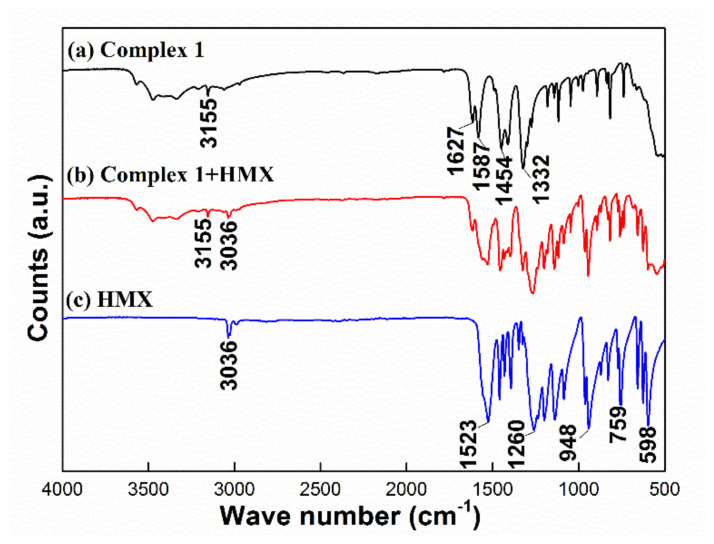
FTIR curves of complex **1** (**a**), complex **1** and HMX (**b**), and HMX (**c**).

In this paper, the compatibility of [La(tza)(NO_3_)_2_(H_2_O)_4_]*_n_* with HMX was further studied using FTIR [35,36]. In Figure 8a, due to the bending vibration and association of O–H from crystalline and coordinated water, complex **1** exhibited a wider absorption peak near 3155 cm^−1^. The asymmetric vibrational C=O peaks near 1627 and 1587 cm^−1^ show that the O atom in C(O)O^−^ was successfully coordinated with the La ion. The absorption peak near 1454 cm^−1^ appeared due to the stretching vibration of C=N in the tetrazole ring. In addition, the absorption peaks near 1332 cm^−1^ indicate that the La metal ion was successfully coordinated with NO_3_^−^ [27]. In Figure 8c, the peaks near 3036 cm^−1^ were due to C–H tensile vibration. The peaks near 1523 cm^−1^ and 759 cm^−1^ were due to –NO_2_ asymmetric, symmetric, and bending vibrations, respectively. The peaks near 1260 cm^−1^ were due to the stretching vibration of N–N [49]. In Figure 8b, no new absorption peak appeared, and the position of the peaks was nearly unchanged compared with those of HMX and complex **1**. The absorption spectrum in Figure 8b was obtained by superimposing Figure 8a,c. Therefore, it was concluded that there was no chemical interaction between the pair of components [35]. Consequently, at room temperature, complex **1** does not interact with HMX, showing that the two compounds have good compatibility.

## 4. Conclusions

Two novel, thermally-stable rare-earth energetic complexes [La(tza)(NO_3_)_2_(H_2_O)_4_]*_n_* and [Ce(tza)(NO_3_)_2_(H_2_O)_4_]_n_ were prepared and evaluated. The two complexes were shown to promote the thermal degradation of HMX. Complex **1** showed a slightly better effect on the thermal decomposition of HMX than **2,** and it was also compatible with HMX. These results show that [La(tza)(NO_3_)_2_(H_2_O)_4_]_n_ may be a more efficient combustion promoter for NEPE propellant. This study could provide beneficial ideas for the rational design and preparation of new energetic promoters for solid propellants.

## Figures and Tables

**Table 1:** Promoting effect of complexes **1** to **5** on the thermal decomposition of HMX [29,45].

Sample	*T*_exo_ (°C)	*H*^1^ (J·g^−1^)	Δ*H* ^2^ (J·g^−1^)	Δ*T* ^3^ (°C)
HMX	282.6	1198.3	-	-
HMX + complex **1**	280.4	1345.7	129.9	2.2
HMX + complex **2**	281.3	1226.4	41.5	1.3
HMX + complex **3**	280.9	1286.3	109.8	1.7
HMX + complex **4**	281.0	1213.2	37.7	1.6
HMX + complex **5**	281.2	1234.3	53.1	1.4

^1^ H: the heat release of HMX or a mixed system. ^2^ ΔH: actual added heat value of the system with complexes **1** to **5**. ^3^ ΔT: the change in the HMX decomposition peak temperature after the addition of complex **1** to **5**.

**Table 2:** Evaluated criterion of compatibility for explosives and energetic materials [36].

Standard (Δ*T*_p_/°C)	Rating		
≦2	A	Compatible or good compatibility	Safe for use in any explosive design.
3–5	B	Slightly sensitized or fair compatibility	Safe for use in testing, when the device is used in a very short period of time; Not to be used as a binder material, or when long-term storage is desired.
6–15	C	Sensitized or poor compatibility	Not recommended for use with explosive items.
>15	D	Hazardous or bad compatibility	Hazardous. Do not use under any conditions.

## Supplementary Materials

The following are available online at https://www.mdpi.com/1996-1944/13/12/2811/s1, Table S1: Crystal data and structure refinement for the complexes **1** and **2**, Table S2: Selected Bond Distances (Å) and Angles (°) for complexes **1** and **2**. The CIF reports of complexes **1** and **2** can be found in the supplementary materials.
