# Supplementary material for: Synthesis and Effects of Two Novel Rare-Earth Energetic Complexes on Thermal Decomposition of Cyclotetramethylene Tetranitramine (HMX)
Source: Materials (Basel). 2020 Jun 22;13(12):2811. doi: 10.3390/ma13122811 (PMC7344791; doi:10.3390/ma13122811)

materials

MDPI

Supplementary Materials

Synthesis and Effects of Two Novel Rare-earth Energetic Complexes on Thermal Decomposition of Cyclotetramethylene tetranitramine (HMX)

Table S1. Crystal data and structure refinement for the complexes 1 and 2.

Complex	1	2
Empirical formula	$C_3H_{11}LaN_6O_{12}$	$C_3H_{11}CeN_6O_{12}$
Formula weight	462.09	463.30
Temperature	100 K	285(2) K
Wavelength	0.71073 Å	0.71073 Å
Crystal system	Orthorhombic	orthorhombic
Space group	P 21 21 21	P 21 21 21
a(Å)	7.9052(6)	7.9022(5)
b(Å)	8.4313(6)	8.4199(5)
c(Å)	19.8024(14)	19.8121(12)
α (°)	90	90
β (°)	90	90
γ (°)	90	90
V(Å3)	1319.85(17)	1318.21(14)
Z	4	4
ϱ(calc.)(Mg/m^3)	2.325	2.334
μ(mm^{-1})	3.318	3.535
F(000)	896	900
Theta range for data collection(°)	2.057 to 27.421	3.18 to 28.28
h/k/l	-10<=h<=10, -10<=k<=10, -25<=l<=25	-6<=h<=10, -11<=k<=11, -26<=l<=26
Reflections collected / unique	11305 /2991 [R(int) = 0.0432]	12798 / 3251 [R(int) =0.0214]
Completeness to theta = 25.02	99.5 %	99.6 %
Data/restraints/parameters	2991 / 8 / 224	3251 / 8 / 126
Goodness-of-fit on F^2	0.985	1.028
Largest diff. peak and hole(e.A^{-3})	0.722 and -0.605	1.147 and -1.462

Table S2. Selected Bond Distances (Å) and Angles (°) for complexes 1and 2.

Complex 1			
La(1)-O(1)	2.723(3)	La(1)-O(2)	2.675(3)
La(1)-O(4)	2.677(3)	La(1)-O(5)	2.648(3)
La(1)-O(7)#1	2.501(3)	La(1)-O(8)	2.538(2)
La(1)-O(9)	2.584(3)	La(1)-O(10)	2.517(3)
La(1)-O(11)	2.580(3)	La(1)-O(12)	2.557(3)
O(2)-La(1)-O(1)	47.12(9)	O(2)-La(1)-O(4)	127.44(9)
O(4)-La(1)-O(1)	154.97(9)	O(5)-La(1)-O(1)	127.36(9)
O(5)-La(1)-O(2)	161.89(10)	O(5)-La(1)-O(4)	47.88(9)
O(7)#1-La(1)-O(1)	73.59(9)	O(7)#1-La(1)-O(2)	118.70(9)
O(7)#1-La(1)-O(4)	112.63(9)	O(7)#1-La(1)-O(5)	65.40(10)

O(7)#1-La(1)-O(8)	76.58(10)	O(7)#1-La(1)-O(9)	147.37(10)
O(7)#1-La(1)-O(10)	140.84(10)	O(7)#1-La(1)-O(11)	73.20(11)
O(7)#1-La(1)-O(12)	76.60(10)	O(8)-La(1)-O(1)	133.83(9)
O(8)-La(1)-O(2)	131.52(10)	O(8)-La(1)-O(4)	70.12(8)
O(8)-La(1)-O(5)	66.03(9)	O(8)-La(1)-O(9)	126.53(10)
O(8)-La(1)-O(11)	71.90(9)	O(8)-La(1)-O(12)	132.68(10)
O(9)-La(1)-O(1)	96.48(10)	O(9)-La(1)-O(2)	66.18(10)
O(9)-La(1)-O(4)	64.81(10)	O(9)-La(1)-O(5)	100.61(10)
O(10)-La(1)-O(1)	112.82(9)	O(10)-La(1)-O(2)	69.39(10)
O(10)-La(1)-O(4)	78.39(10)	O(10)-La(1)-O(5)	119.81(10)
O(10)-La(1)-O(8)	72.36(10)	O(10)-La(1)-O(9)	71.77(10)
O(10)-La(1)-O(11)	74.89(10)	O(10)-La(1)-O(12)	142.54(10)
O(11)-La(1)-O(1)	66.21(10)	O(11)-La(1)-O(2)	70.27(10)
O(11)-La(1)-O(4)	138.57(10)	O(11)-La(1)-O(5)	125.93(10)
O(11)-La(1)-O(9)	131.98(11)	O(12)-La(1)-O(1)	72.14(10)
O(12)-La(1)-O(2)	95.60(10)	O(12)-La(1)-O(4)	85.47(10)
O(12)-La(1)-O(5)	67.52(10)	O(12)-La(1)-O(9)	70.77(11)
O(12)-La(1)-O(11)	133.74(10)	N(1)-O(1)-La(1)	96.2(2)
N(1)-O(2)-La(1)	98.6(2)	N(2)-O(4)-La(1)	96.7(2)
N(2)-O(5)-La(1)	97.5(2)	C(1)-O(7)-La(1)#2	150.1(3)
C(1)-O(8)-La(1)	136.9(3)	La(1)-O(1)-N(1)-O(2)	9.3(4)
La(1)-O(1)-N(1)-O(3)	-172.0(3)	La(1)-O(2)-N(1)-O(1)	-9.6(4)
La(1)-O(2)-N(1)-O(3)	171.7(3)	La(1)-O(4)-N(2)-O(5)	9.2(4)
La(1)-O(4)-N(2)-O(6)	-170.8(3)	La(1)-O(5)-N(2)-O(4)	-9.3(4)
La(1)-O(5)-N(2)-O(6)	170.7(3)		
Symmetry transformations used to generate equivalent atoms: #1 -x,y-1/2,-z+1/2; #2 -x,y+1/2,-z+1/2			

**Complex 2**			
O(2W)-Ce(1)	2.564(3)	O(1W)-Ce(1)	2.537(3)
O(3)-Ce(1)	2.623(3)	O(3W)-Ce(1)	2.496(3)
O(6)-Ce(1)	2.673(3)	O(7)-Ce(1)	2.700(3)
O(4W)-Ce(1)	2.571(3)	O(2)-Ce(1)#1	2.524(3)
O(4)-Ce(1)	2.666(3)	Ce(1)-O(1)	2.482(3)
Ce(1)-O(2)#2	2.524(3)		
N(5)-O(3)-Ce(1)	97.9(2)	N(6)-O(6)-Ce(1)	98.0(2)
N(6)-O(7)-Ce(1)	96.8(2)	C(3)-O(2)-Ce(1)#1	137.4(2)
N(5)-O(4)-Ce(1)	96.2(3)	N(5)-O(4)-Ce(1)	96.2(3)
O(1)-Ce(1)-O(3W)	141.45(9)	O(1)-Ce(1)-O(2)#2	76.75(9)
O(3W)-Ce(1)-O(2)#2	72.32(10)	O(1)-Ce(1)-O(1W)	76.53(10)
O(3W)-Ce(1)-O(1W)	142.00(10)	O(3W)-Ce(1)-O(1W)	142.00(10)
O(2)#2-Ce(1)-O(1W)	133.38(10)	O(1)-Ce(1)-O(2W)	73.61(10)
O(3W)-Ce(1)-O(2W)	75.16(10)	O(2)#2-Ce(1)-O(2W)	71.55(10)
O(1W)-Ce(1)-O(2W)	133.72(11)	O(1)-Ce(1)-O(4W)	147.11(10)
O(3W)-Ce(1)-O(4W)	71.43(10)	O(2)#2-Ce(1)-O(4W)	127.10(10)
O(1W)-Ce(1)-O(4W)	70.58(10)	O(2W)-Ce(1)-O(4W)	131.44(11)
O(1)-Ce(1)-O(3)	65.45(10)	O(3W)-Ce(1)-O(3)	119.36(10)
O(2)#2-Ce(1)-O(3)	66.29(9)	O(1W)-Ce(1)-O(3)	68.02(10)
O(2W)-Ce(1)-O(3)	126.11(10)	O(4W)-Ce(1)-O(3)	100.98(10)
O(1)-Ce(1)-O(4)	112.99(9)	O(1)-Ce(1)-O(4)	112.99(9)
O(3W)-Ce(1)-O(4)	77.51(10)	O(2)#2-Ce(1)-O(4)	70.40(9)

O(1W)-Ce(1)-O(4)	86.07(10)	O(2W)-Ce(1)-O(4)	138.25(10)
O(4W)-Ce(1)-O(4)	65.12(10)	O(3)-Ce(1)-O(4)	48.17(10)
O(1)-Ce(1)-O(6)	118.60(10)	O(3W)-Ce(1)-O(6)	69.71(10)
O(2)#2-Ce(1)-O(6)	131.35(10)	O(1W)-Ce(1)-O(6)	95.05(10)
O(2W)-Ce(1)-O(6)	70.21(10)	O(4W)-Ce(1)-O(6)	65.70(10)
O(3)-Ce(1)-O(6)	161.81(10)	O(4)-Ce(1)-O(6)	127.17(10)
O(1)-Ce(1)-O(7)	73.65(10)	O(3W)-Ce(1)-O(7)	113.22(10)
O(2)#2-Ce(1)-O(7)	133.85(9)	O(1W)-Ce(1)-O(7)	71.55(10)
O(2W)-Ce(1)-O(7)	66.60(11)	O(4W)-Ce(1)-O(7)	95.74(10)
O(3)-Ce(1)-O(7)	127.42(10)	O(4)-Ce(1)-O(7)	154.80(10)
O(6)-Ce(1)-O(7)	46.95(10)		

Hydrogen Bond Interactions in Complex 2			
**D-H···A**	**D···A(Å)**	**D-H···A**	D···A(Å)
O3W-H3WA···O2	2.677	O2W-H2WA···O1	2.609
O4W-H4WB···N6	2.706	O3W-H3WA···O1	2.147
O2W-H2WA···O3	2.095	O3W-H3WB···N4	1.959
O4W-H4WA···O8	2.374	O1W-H1WB···N2	2.192
O4W-H4WB···O8	2.146	O1W-H1WA···N2	2.852
O3W-H3WA···O2	2.758	O2W-H2WB···O5	2.011
O2W-H2WB···O4	2.692	O4W-H4WB···O6	2.600
O3W-H3WB···N3	2.876	O2W-H2WB···O1	2.813
O1W-H1WA···N3	2.070		
Symmetry transformations used to generate equivalent atoms:#1 -x+1,y+1/2,-z+3/2；#2 -x+1,y-1/2,-z+3/2			

CIF Report 1

Structure factors have been supplied for datablock(s) w

THIS REPORT IS FOR GUIDANCE ONLY. IF USED AS PART OF A REVIEW PROCEDURE FOR PUBLICATION, IT SHOULD NOT REPLACE THE EXPERTISE OF AN EXPERIENCED CRYSTALLOGRAPHIC REFEREE.

No syntax errors found. CIF dictionary Interpreting this report

Datablock: w

Bond precision:	C-C = 0.0060 A	Wavelength=0.71073	
Cell:	a=7.9052(6)	b=8.4313(6)	c=19.8024(14)
	alpha=90	beta=90	gamma=90
Temperature:	100 K		
	Calculated	Reported	
Volume	1319.85(17)	1319.85(17)	
Space group	P 21 21 21	P 21 21 21	
Hall group	P 2ac 2ab	P 2ac 2ab	
Moiety formula	C3 H11 La N6 O12	C3H11 La N6 O12	
Sum formula	C3 H11 La N6 O12	C3 H11 La N6 O12	
Mr	462.09	462.09	
Dx,g cm-3	2.326	2.325	
Z	4	4	
F000	896.0	896.0	
F000’	895.37		

h,k,lmax	10,10,25	10,10,25
Nref	3007[1748]	2991
Tmin,Tmax		0.472,0.746
Tmin'		

Correction method= # Reported T Limits: Tmin=0.472 Tmax=0.746
AbsCorr = MULTI-SCAN

Data completeness= 1.71/0.99	Theta(max)= 27.421	
R(reflections)= 0.0197(2930)	wR2(reflections)= 0.0395(2991)	
S = 0.985	Npar= 224	

The following ALERTS were generated. Each ALERT has the format
test-name_ALERT_alert-type_alert-level .
Click on the hyperlinks for more details of the test.

Alert level C

PLAT053_ALERT_1_C Minimum Crystal Dimension Missing (or Error) ... Please Check
PLAT054_ALERT_1_C Medium Crystal Dimension Missing (or Error) ... Please Check
PLAT055_ALERT_1_C Maximum Crystal Dimension Missing (or Error) ... Please Check
PLAT090_ALERT_3_C Poor Data / Parameter Ratio (Zmax > 18) .... 7.77 Note
PLAT911_ALERT_3_C Missing # FCF Refl Between THmin & STh/L= 0.600 7 Report
PLAT978_ALERT_2_C Number C-C Bonds with Positive Residual Density. 0 Note

Alert level G

PLAT002_ALERT_2_G Number of Distance or Angle Restraints on AtSite 12 Note
PLAT004_ALERT_5_G Polymeric Structure Found with Maximum Dimension 1 Info
PLAT172_ALERT_4_G The CIF-Embedded .res File Contains DFIX Records 4 Report
PLAT232_ALERT_2_G Hirshfeld Test Diff (M-X) La1 -- O1 .. 6.3 s.u.
PLAT860_ALERT_3_G Number of Least-Squares Restraints .... 8 Note
PLAT913_ALERT_3_G Missing # of Very Strong Reflections in FCF .... 2 Note
PLAT933_ALERT_2_G Number of OMIT Records in Embedded .res File ... 4 Note

0 ALERT level A = Most likely a serious problem - resolve or explain
0 ALERT level B = A potentially serious problem, consider carefully
6 ALERT level C = Check. Ensure it is not caused by an omission or oversight
7 ALERT level G = General information/check it is not something unexpected
3 ALERT type 1 CIF construction/syntax error, inconsistent or missing data
4 ALERT type 2 Indicator that the structure model may be wrong or deficient
4 ALERT type 3 Indicator that the structure quality may be low
1 ALERT type 4 Improvement, methodology, query or suggestion
1 ALERT type 5 Informative message, check

It is advisable to attempt to resolve as many as possible of the alerts in all categories. Often the minor alerts point to easily fixed oversights, errors and omissions in your CIF or refinement strategy, so attention to these fine details can be worthwhile. In order to resolve some of the more serious problems it may be necessary to carry out additional measurements or structure refinements. However, the purpose of your study may justify the reported deviations and the more serious of these should normally be commented upon in the discussion or experimental section of a paper or in

the "special_details" fields of the CIF. Check CIF was carefully designed to identify outliers and unusual parameters, but every test has its limitations and alerts that are not important in a particular case may appear. Conversely, the absence of alerts does not guarantee there are no aspects of the results needing attention. It is up to the individual to critically assess their own results and, if necessary, seek expert advice.

Publication of your CIF in IUCr journals

A basic structural check has been run on your CIF. These basic checks will be run on all CIFs submitted for publication in IUCr journals (Acta Crystallographica, Journal of Applied Crystallography, Journal of Synchrotron Radiation); however, if you intend to submit to Acta Crystallographica Section C or E or IUCrData, you should make sure that full publication checks are run on the final version of your CIF prior to submission.

Publication of your CIF in other journals

Please refer to the Notes for Authors of the relevant journal for any special instructions relating to CIF submission.

PLATON version of 27/03/2017; check.def file version of 24/03/2017

Datablock w - ellipsoid plot

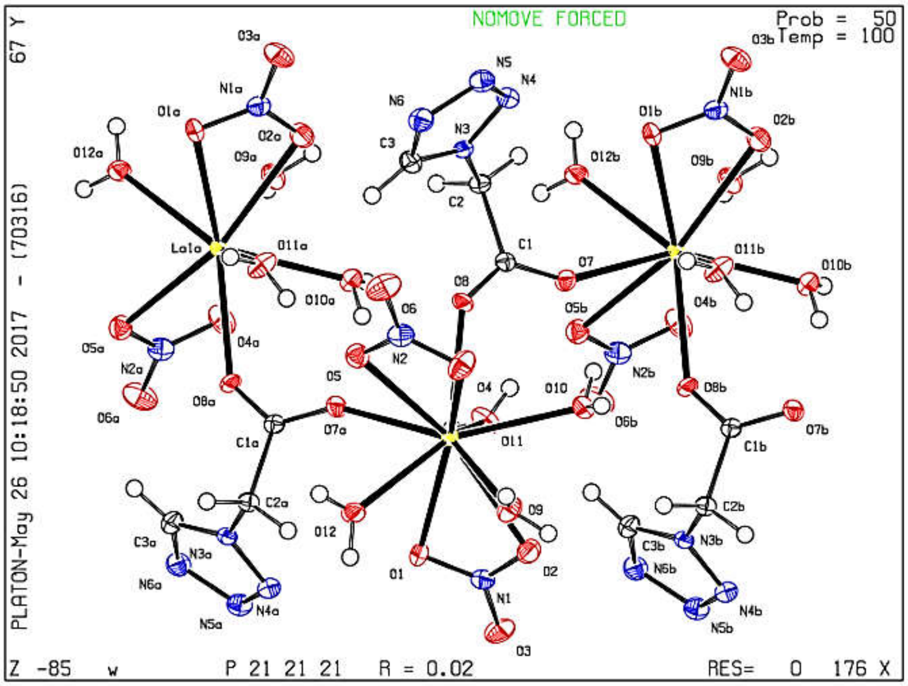

CIF Report 2

Datablock: Complex 2

Bond precision:	C-C = 0.0020 A		Wavelength=0.71073
Cell:	a=7.9022(5)	b=8.4199(5)	c=19.8121(12)
	alpha=90	beta=90	gamma=90

Temperature: 293 K

	Calculated	Reported
Volume	1318.21(14)	1318.21(14)
Space group	P 21 21 21	P2(1)2(1)2(1)
Hall group	P 2ac 2ab	P 2ac 2ab
Moiety formula	C3 H11 Ce N6 O12	C3 H11 Ce N6 O12
Sum formula	C3 H11 Ce N6 O12	C3 H11 Ce N6 O12
Mr	463.30	463.30
Dx,g cm-3	2.335	2.334
Z	4	4
Mu (mm-1)	3.535	3.535
F000	900.0	900.0
F000'	899.52	
h,k,lmax	10,11,26	10,11,26
Nref	3272[1897]	3251
Tmin,Tmax	0.291,0.428	0.363,0.484
Tmin'	0.269	

Correction method= # Reported T Limits: Tmin=0.363 Tmax=0.484 AbsCorr = EMPIRICAL

Data completeness= 1.71/0.99 Theta(max)= 28.280

R(reflections)= 0.0111(3225) wR2(reflections)= 0.0273(3251)

S = 1.097 Npar= 236

The following ALERTS were generated. Each ALERT has the format

test-name_ALERT_alert-type_alert-level.

Click on the hyperlinks for more details of the test.

Alert level C

ABSTY02_ALERT_1_C An _exptl_absorpt_correction_type has been given without a literature citation. This should be contained in the _exptl_absorpt_process_details field.

Absorption correction given as Empirical

PLAT094_ALERT_2_C Ratio of Maximum / Minimum Residual Density .... 2.05 Report

PLAT230_ALERT_2_C Hirshfeld Test Diff for O4 --N5 . 5.5 s.u.

PLAT241_ALERT_2_C High 'MainMol' Ueq as Compared to Neighbors of O4 Check

---

Alert level G

PLAT002_ALERT_2_G Number of Distance or Angle Restraints on AtSite 12 Note

PLAT004_ALERT_5_G Polymeric Structure Found with Maximum Dimension 1 Info

PLAT005_ALERT_5_G No Embedded Refinement Details Found in the CIF Please Do !

PLAT164_ALERT_4_G Nr. of Refined C-H H-Atoms in Heavy-Atom Struct. 1 Note

PLAT199_ALERT_1_G Reported _cell_measurement_temperature ..... (K) 293 Check
PLAT200_ALERT_1_G Reported _diffrn_ambient_temperature ..... (K) 293 Check
PLAT232_ALERT_2_G Hirshfeld Test Diff (M-X) Ce1 --O1W . 6.0 s.u.

And 7 other PLAT232 Alerts

More ...

PLAT720_ALERT_4_G Number of Unusual/Non-Standard Labels .... 8 Note
PLAT794_ALERT_5_G Tentative Bond Valency for Ce1 (III) . 3.18 Inf
PLAT860_ALERT_3_G Number of Least-Squares Restraints .... 8 Note
PLAT899_ALERT_4_G SHELXL97 is Deprecated and Succeeded by SHELXL 2018 Note

---

0 **ALERT level A** = Most likely a serious problem - resolve or explain
0 **ALERT level B** = A potentially serious problem, consider carefully
4 **ALERT level C** = Check. Ensure it is not caused by an omission or oversight
18 **ALERT level G** = General information/check it is not something unexpected

3 ALERT type 1 CIF construction/syntax error, inconsistent or missing data
12 ALERT type 2 Indicator that the structure model may be wrong or deficient
1 ALERT type 3 Indicator that the structure quality may be low
3 ALERT type 4 Improvement, methodology, query or suggestion
3 ALERT type 5 Informative message, check

It is advisable to attempt to resolve as many as possible of the alerts in all categories. Often the minor alerts point to easily fixed oversights, errors and omissions in your CIF or refinement strategy, so attention to these fine details can be worthwhile. In order to resolve some of the more serious problems it may be necessary to carry out additional measurements or structure refinements. However, the purpose of your study may justify the reported deviations and the more serious of these should normally be commented upon in the discussion or experimental section of a paper or in the "special_details" fields of the CIF. checkCIF was carefully designed to identify outliers and unusual parameters, but every test has its limitations and alerts that are not important in a particular case may appear. Conversely, the absence of alerts does not guarantee there are no aspects of the results needing attention. It is up to the individual to critically assess their own results and, if necessary, seek expert advice.

Publication of your CIF in IUCr journals

A basic structural check has been run on your CIF. These basic checks will be run on all CIFs submitted for publication in IUCr journals (*Acta Crystallographica, Journal of Applied Crystallography, Journal of Synchrotron Radiation*); however, if you intend to submit to *Acta Crystallographica Section C* or *E* or *IUCrData*, you should make sure that full publication checks are run on the final version of your CIF prior to submission.

Publication of your CIF in other journals

Please refer to the *Notes for Authors* of the relevant journal for any special instructions relating to CIF submission.

PLATON version of 20/08/2018; check.def file version of 20/08/2018

Datablock 2 - ellipsoid plot

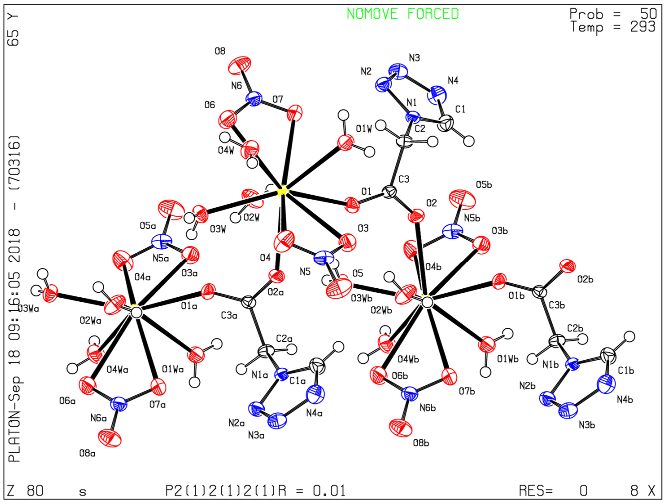

Supplement: Supplementary file 1 [file materials-13-02811-s001.pdf]
